# Preoperative SCC-Ag as a predictive marker for the use of adjuvant chemotherapy in cervical squamous cell carcinoma with intermediate-risk factors

**DOI:** 10.1186/s12885-020-06928-9

**Published:** 2020-05-19

**Authors:** Hong-tao Guo, Xue-han Bi, Ting Lei, Xiao Lv, Guang Yao, Yao Chen, Chang Liu

**Affiliations:** Department of obstetrics and gynecology, the First Hospital of Lanzhou University, Key Laboratory of Gynecologic Oncology Gansu Province, No.1, Donggang West Road, Lanzhou, 730000 Gansu Province China

**Keywords:** Cervical cancer, Chemotherapy, Radiotherapy, Squamous cell carcinoma antigen

## Abstract

**Background:**

For cervical cancer patients whose tumors display a combination of intermediate risk factors, postoperative radiation with or without adjuvant chemotherapy is suggested for them. However, who should be administered with adjuvant chemotherapy is unknown. The current study was designed to explore the clinical value of squamous cell carcinoma antigen (SCC-Ag) in guiding the use of adjuvant chemotherapy in cervical cancer patients.

**Methods:**

A total of 301 cervical cancer patients were included in the present study from March 2006 to March 2016. There were 156 patents who received adjuvant chemotherapy, while the rest of 145 patents did not receive it. The survival analysis including Overall survival (OS) and disease-free survival (DFS) was assessed by using the Kaplan-Meier method. Cox proportional hazards regression was done to detect factors in predicting the tumor prognosis.

**Results:**

In patients with high pre-treatment SCC-Ag level, those who received adjuvant chemotherapy acquired better prognosis than patients who did not receive it. Particularly, a lower rate of distant metastasis was found in the group of adjuvant chemo-radiotherapy than that in the group of adjuvant radiotherapy. As for patients with low pre-treatment SCC-Ag level, we observed no differences in both the OS and DFS between patients who were given and not given with adjuvant chemotherapy. In the multivariable analysis, adjuvant chemotherapy was significantly correlated with DFS and distant metastasis-free survival (DMFS) in patients with high SCC-Ag level.

**Conclusion:**

Preoperative SCC-Ag can be a predictive marker for the use of adjuvant chemotherapy in cervical squamous cell carcinoma with intermediate-risk factors.

## Background

As we know, cervical cancer is the one of the most common cancer in women worldwide [1]. The standard treatment for early-stage cervical cancer is surgery. However, adjuvant chemo-radiotherapy plays still an important role in the integrated therapy when some pathological findings are found after surgery. The common factors such as lymph node metastasis, parametrial involvement and positive surgical margin are known as “high-risk” factors and patients with any of these features are suggested to receive postoperative concurrent chemo-radiotherapy [2]. But, for patients whose tumors present with a combination of intermediate risk factors such as large size, deep stromal invasion, and lymphovascular involvement, postoperative pelvic radiation is suggested for them with no mandatory need of adjuvant chemotherapy [3]. Thus, the problem is that who should be given with adjuvant chemotherapy is still unknown for the patients with two intermediate-risk factors. Moreover, there is also no consensus reached by physicians on this topic, leading to over or less treatment for some patients with intermediate-risk factors.

Squamous cell carcinoma antigen (SCC-Ag), which is produced through squamous formation of cervical squamous epithelium, is a biomarker routinely used in clinical practice [4]. Approximately 28 to 88% of cervical squamous cell carcinomas were with abnormal level of SCC-Ag level, which is very meaningful in cervical cancer patients [5]. Many studies found that pre-treatment SCC-Ag level could predict disease progression after treatments [6–12]. Besides, SCC-Ag was also employed to evaluate the response to treatment [13]. However whether preoperative SCC-Ag can be a predictive marker for the use of adjuvant chemotherapy in cervical squamous cell carcinoma is still unknown. In our present study, we tried to identify the clinical value of SCC-Ag in the administration of adjuvant chemotherapy in early stage cervical cancer with intermediate-risk factors. Our findings indicated that there was no need to administer adjuvant chemotherapy to patients with low preoperative SCC-Ag level. While, it was beneficial for patients with high SCC-Ag level to receive adjuvant chemotherapy. We just presented a novel use of SCC-Ag to be a marker for the effectiveness of adjuvant chemotherapy in the clinical practice. It could be one of the first articles on the use of preoperative SCC-Ag in guiding the administration of adjuvant chemotherapy in patients with cervical cancer since few researches have investigated the relationship between SCC-Ag level and the use of adjuvant chemotherapy in cervical cancer patients with intermediate-risk factors.

## Methods

### Ethics statement

This research was approved by the First Hospital of Lanzhou University, and written informed consent was obtained from every patient included in the study.

### Patients and procedures

We acquired our data from a database at First Hospital of Lanzhou University from March 2006 to March 2016. The selection criteria for the current study were as follows: (1) pathologically confirmed uterine cervical cancer with two intermediate-risk factors; (2) received surgery followed by adjuvant radiotherapy or adjuvant chemo-radiotherapy; (3) the function of liver and renal function is normal; (4) no concurrent cancer and (5) did not receive radiotherapy to the pelvis previously. Patients with any high-risk factors were excluded. After careful reviewing the patients’ information, 301 patients met the inclusion criteria and were analyzed in the present study.

### Clinical evaluation

We performed the clinical staging with the help of physical examination, computed tomography or magnetic resonance imaging, and chest radiography. Besides, complete blood count and liver function test were also performed. Pre-treatment SCC-Ag levels were measured within 2 weeks before surgery. As for the method to measure serum SCC-Ag levels, we adopted sandwich enzyme linked immunosorbent assay (ELISA) technique by using ELISA Kit. In brief, 4-5 mL venous blood samples were collected form the patients and centrifuged. First, we prepared the ELISA plates which were coated with an antibody specific to SCC-Ag. Then, the standards and the samples were added to the ELISA plate wells. After incubation for 90 mins, a horseradish peroxidase-conjugated polyclonal antibody specific for SCC-Ag was added to each well to “sandwich” the SCC-Ag. Then, the plate was incubated for 30 mins and washed with wash buffer to remove components which were uncombined. Next, the substrate solution was added to each well, followed by a short period of incubation for 15 mins. The wells which contained SCC-Ag would present a color change. Finally, sulfuric acid solution was used to stop the enzyme-substrate reaction and we measured the color change by the method of spectrophotometry. The SCC-Ag concentration in each sample was estimated from the standard curve established based on the concentration of standards. All the patients included in our study were with elevated pre-treatment SCC-Ag levels (Range 2.21–45.57 ng/mL). (In our hospital, the normal level of SCC in healthy individuals is less than 2.00 ng/mL). The median level of SCC-Ag for the whole group of patients was 6.09 ng/mL. And we adopted the median level of SCC-Ag to divide all the patients into two groups: high squamous cell carcinoma level group (> 6.09 ng/mL) and low squamous cell carcinoma level group (≤6.09 ng/mL). The tumor size of 4 cm was used as a cutoff value to differentiate tumor size and as a predictor of oncologic outcome according to the previously published researches [14, 15].

### Chemotherapy

Part of the patients received adjuvant chemotherapy. The regimen usually contained 5-Fu (3-4 g/m^2^, civ96h) and cisplatin (70 mg/m^2^) and it was given to the patients every 3 weeks. Besides, other regimen including paclitaxel plus cisplatin was also used. And the details of this regimen are as follows: paclitaxel 135 mg/m^2^ and cisplatin 70 mg/m^2^. The median cycles of adjuvant chemotherapy were 3 (2–4). Usually, two cycles of adjuvant chemotherapy were concurrent with postoperative radiotherapy.

### Radiotherapy

Postoperative radiotherapy was scheduled for all the patients. The radiation dose for the whole pelvis was 45–50 Gy/23-25F. Radiotherapy was performed for 5 days per week with a total treatment duration of 5–6 weeks. In making the plan of radiotherapy, the clinical target volume (CTV) should encompass the tumor bed and the associated pelvic lymphatic drainage area such as common iliac lymph nodes, internal and external iliac lymph nodes, as well as the sacral lymph nodes. The supra-vaginal portion should also be included in the CTV. The bottom of L4 was defined as superior border of the CTV. While, the lower margin of the obturator was regarded as the inferior border of CTV. The anterior and posterior borders of CTV were the posterior wall of urinary bladder and anterior margin of the sacrum, respectively.

### Follow-up evaluation

The follow up policy was as following: for the first 2 years, patients should be evaluated every 3 months. After 2 years, patients can be followed up every 6 months. When the total follows up time exceeds 5 years, patients were recommended to receive medical examination annually. The evaluation usually included blood related tests such as blood cell counts, SCC-Ag et al. Patients also took the examinations of computed tomography or magnetic resonance imaging of the abdomen and pelvis every 6 months. Besides, chest radiography was also suggested for them during each visit. The DFS and OS in the present study were defined from the date of diagnosis to the date of recurrence and to the date of death, respectively. While, for patients who showed no death or recurrence, the date of last follow-up was defined as OS and DFS.

### Statistical analysis

The statistical analyses were done by using SPSS software, version 20.0. Categorical variables were analyzed using the chi-square test or Fisher’s exact test. Continuous variables were analyzed using the Student’s t test or the Mann–Whitney U test. The comparisons of disease-free survival and overall survival rates between different group were performed by using Kaplan–Meier method. Multivariate analysis of disease-free survival, local recurrence-free survival and distant metastasis-free survival was analyzed using Cox proportional hazards regression. *P* < 0.05 was considered to be statistically significant.

## Results

### Clinical characteristics

In all, we enrolled 301 cervical cancer patients who were with two intermediate risk factors. Among them, 156 patents received adjuvant chemo-radiotherapy, while the rest of 145 patents received adjuvant radiotherapy alone. Compared to patients who received adjuvant radiotherapy alone, those who received chemo-radiotherapy presented no difference in clinical tumor stage, tumor size, lympho-vascular involvement, deep stromal invasion and follow-up. However, patients who did receive adjuvant concurrent chemotherapy tend to be younger than those who did not (Table 1).

**Table 1:** Patient Demographics and Baseline Tumor Characteristics

Variable	Adjuvant chemo-radiotherapy (*n* = 156)	Adjuvant radiotherapy (*n* = 145)	*p* value
Age, year			0.046
median	60	63	
Stage			0.752
IA2	4	5	
IB1	88	82	
IB2	11	13	
IIA1	36	35	
IIA2	17	10	
LVSI			0.562
yes	66	67	
no	90	78	
DSI			1.000
yes	132	122	
no	24	23	
Tumor size			0.526
≥ 4	114	101	
< 4	42	44	
Risk group			0.787
large Tumor+DSI	90	78	
large Tumor+LVSI	24	23	
DSI + LVSI	42	44	
Follow up, months			0.932
median	56.9	56.6	

Abbreviation: *DSI* deep stromal invasion, *LVSI* lymph-vascular space invasion, *SCC* squamous cell carcinoma

### Survival analysis for the whole group

**Fig. 1:**
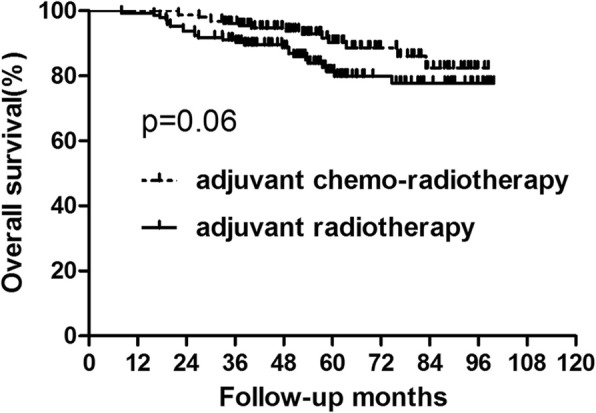
Overall survival for the whole group of patients. No significant difference was found in overall survival between patients who did and did not receive concurrent chemotherapy (*P* = 0.060)

During the follow up, for the whole group, there were 40 patients who died. The 5-year overall survival in the adjuvant chemo-radiotherapy and adjuvant radiotherapy groups were 90.29 and 81.29%, respectively (Fig. 1, Table 2). No significant difference was showed in overall survival between the two groups Fifty-six patients suffered recurrence, of them, local recurrence was found in 13 patients, distant metastasis was showed in 28 patients and 15 patients were with both local and distant recurrences. The common metastatic sites were liver, lung, bone and lymph nodes. Compared to patients who did not received adjuvant chemotherapy, those who did acquired better disease-free survival (86.11% vs 74.89%, *p* = 0.004) (Fig. 2, Table 2).

**Table 2:** Survival for the Whole Group Patients

Group	Adjuvant chemo-radiotherapy (*n* = 156)		Adjuvant radiotherapy (*n* = 145)		*P* value
	3-year	5-year	3-year	5-year	
OS	96.15%	90.29%	91.02%	81.29%	0.060
DFS	91.59%	86.11%	80.67%	74.89%	0.004

Abbreviations: *OS* overall survival, *DFS* disease-free survival

#: calculated by Kaplan–Meier method

**Fig. 2:**
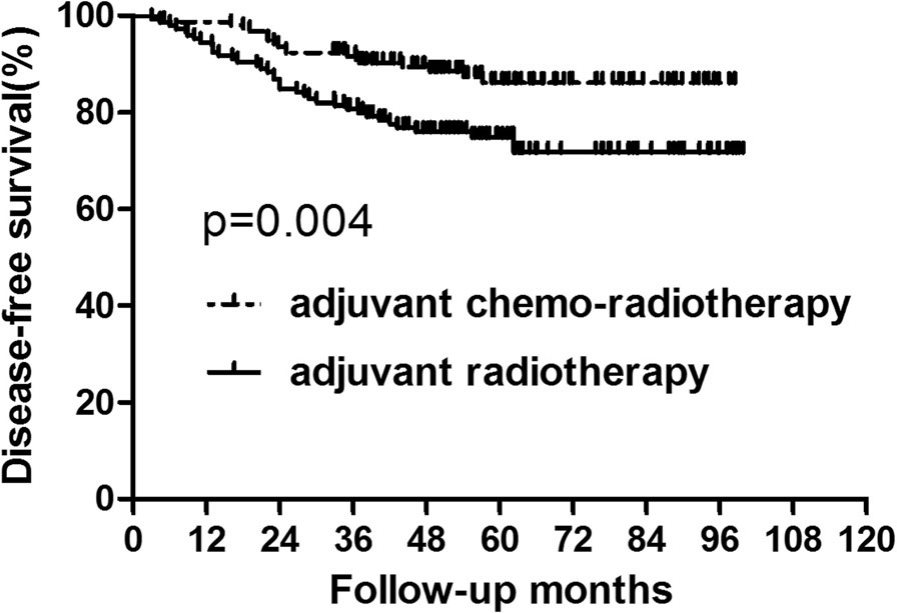
Disease-free survival for the whole group of patients. Significant difference was found in disease-free survival between patients who did and did not receive concurrent chemotherapy (*P* = 0.004)

### Survival analysis for patients with high squamous cell carcinoma level

**Fig. 3:**
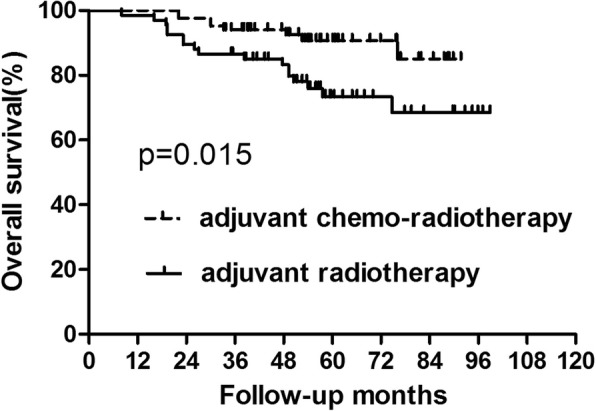
Overall survival for the subgroup of patients with high squamous cell carcinoma level. Significant difference was found in overall survival between patients who did and did not receive concurrent chemotherapy (*P* = 0.015)

**Fig. 4:**
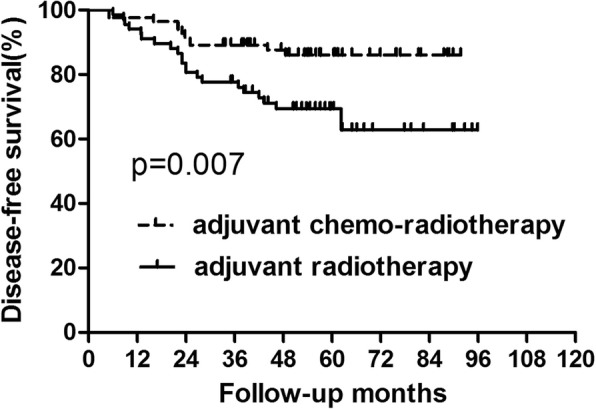
Disease-free survival for the subgroup of patients with high squamous cell carcinoma level. Significant difference was found in disease-free survival between patients who did and did not receive concurrent chemotherapy (*P* = 0.007)

For patients with high SCC-Ag level, there were 25 cases who died and there were 33 patients who developed recurrence. Nine patients were with local recurrence alone and 13 patients suffered from only distant metastasis. Additionally, 11 patients presented with both local and distant metastasis. Patients in the adjuvant chemo-radiotherapy group acquired better 5-year OS (90.72% vs73.41%, *p* = 0.015) and DFS (86.03% vs 69.40%, *p* = 0.007) than those in the adjuvant radiotherapy group (Figs. 3 and 4, Table 3). We also analyze the recurrence pattern, with result showing that there was no difference in local recurrence between groups with radiotherapy and chem-radiotherapy. However, distant metastasis was significantly higher in the radiotherapy group than that in the chemo-radiotherapy group (*p* = 0.002) (Table 4).

**Table 3:** Survival for the Patients with high SCC level

Group	Adjuvant chemo-radiotherapy (*n* = 84)		Adjuvant radiotherapy (*n* = 67)		*p* value
	3-year	5-year	3-year	5-year	
OS	94.05%	90.72%	86.57%	73.41%	0.015
DFS	89.16%	86.03%	77.61%	69.40%	0.007

Abbreviations: *OS* overall survival, *DFS* disease-free survival

#: calculated by Kaplan–Meier method

**Table 4:** Recurrence Patterns for Patients with high SCC level

Group	Adjuvant chemo-radiotherapy (*n* = 84)		Adjuvant radiotherapy (*n* = 67)		*p* value
	3-year	5-year	3-year	5-year	
LR	5 (6.0%)	7 (8.3%)	8 (11.9%)	10 (14.9%)	0.069
SM	5 (6.0%)	6 (7.1%)	11 (16.4%)	18 (26.9%)	0.002

Abbreviations: *LR* local recurrence, *SM* systemic metastases

#: calculated by Kaplan–Meier method

### Survival analysis for patients with low squamous cell carcinoma level

**Fig. 5:**
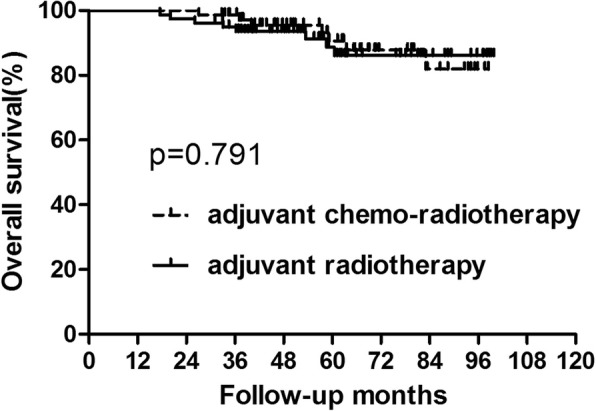
Overall survival for the subgroup of patients with low squamous cell carcinoma level. No significant difference was found in overall survival between patients who did and did not receive concurrent chemotherapy (*P* = 0.791)

**Fig. 6:**
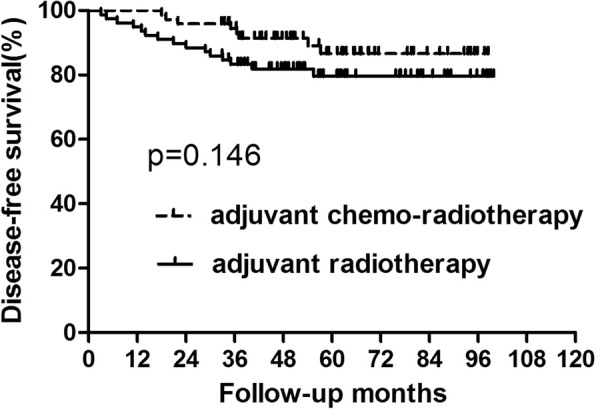
Disease-free survival for the subgroup of patients with low squamous cell carcinoma level. No significant difference was found in disease-free survival between patients who did and did not receive concurrent chemotherapy (*P* = 0.146)

For patients with low SCC-Ag level, 23 patients recurred with 15 patents dying of tumor recurrence. Four patients recurred only locally, 15 patients had only distant metastasis and 4 patients developed both local and distant recurrences. The 5-year OS in the adjuvant chemo-radiotherapy and adjuvant radiotherapy groups was 90.65 and 88.74%, respectively (Fig. 5, Table 5). The 5-year DFS in these two groups was 86.62 and 79.63%, respectively (Fig. 6, Table 5). No significant differences were found in both OS (*p* = 0.097) and DFS (*p* = 0.253). Further analysis of recurrence pattern results just showed that there were no differences in both the local and distant failure between patients did and did not receive adjuvant chemotherapy (Table 6).

**Table 5:** Survival for the Patients with low SCC level

Group	Adjuvant chemo-radiotherapy (*n* = 72)		Adjuvant radiotherapy (*n* = 78)		*p* value
	3-year	5-year	3-year	5-year	
OS	98.61%	90.65%	94.84%	88.74%	0.791
DFS	94.36%	86.62%	83.27%	79.63%	0.146

Abbreviations: *OS* overall survival, *DFS* disease-free survival

#: calculated by Kaplan–Meier method

**Table 6:** Recurrence Patterns for Patients with low SCC level

Group	Adjuvant chemo-radiotherapy (*n* = 72)		Adjuvant Radiotherapy (*n* = 78)		*p* value
	3-year	5-year	3-year	5-year	
LR	1 (1.4%)	3 (4.2%)	2 (2.6%)	5 (6.4%)	0.612
SM	3 (4.2%)	8 (11.1%)	10 (12.8%)	11 (14.1%)	0.515

Abbreviations: *LR* local recurrence, *SM* systemic metastases

#: calculated by Kaplan–Meier method

### Clinical predictors for disease-free survival, local recurrence-free survival and distant metastasis-free survival for patients with high squamous cell carcinoma level

For patients with high SCC-Ag level, results showed that tumor size and adjuvant chemotherapy were independent predictors of DFS and DMFS. Besides, adjuvant chemotherapy was found to be the unique factor significantly associated with DMFS, indicating that patients who received adjuvant chemotherapy suffered less distant failure than those who did not (Table 7).

**Table 7:** Multivariate Analyses of DFS, LRFS, and DMFS for Patients with high SCC level

Variable	DFS		LRFS		DMFS	
	HR (95%CI)	*p* value	HR (95%CI)	*p* value	HR (95%CI)	*p* value
Adjuvant chemotherapy Yes vs no	0.456 (0.217–0.957)	0.038	0.538 (0.207–1.401)	0.204	0.282 (0.111–0.721)	0.008
Tumor size ≥4 cm vs < 4 cm	2.988 (1.278–6.984)	0.012	3.213 (1.426–7.335)	0.007	1.731 (0.701–4.276)	0.234
DSI Yes vs no	2.083 (0.988–4.391)	0.054	2.886 (1.012–8.235)	0.048	1.410 (0.603–3.296)	0.428
LVSI No vs yes	0.645 (0.319–1.302)	0.221	0.610 (0.241–1.541)	0.296	0.658 (0.288–1.501)	0.319

Abbreviations: *DFS* disease-free survival, *LRFS* local recurrence-free survival, *DMFS* distant metastasis-free survival, *DSI* deep stromal invasion, *LVSI* lymph-vascular space invasion, *SCC* squamous cell carcinoma;

## Discussion

Our current study demonstrated that, for patients with intermediate-risk factors, those who received adjuvant chemotherapy acquired better DFS than those who did not, although no significant differences was found in OS. Based on the pre-treatment SCC-Ag level, we further performed subgroup analysis with results showing that adjuvant chemotherapy was clinically meaningful only in patients with elevated SCC-Ag level by improving both the DFS and OS. However, in patients with low SCC-Ag level, adjuvant concurrent chemotherapy seemed to contribute little in improving the survival in this subgroup. Additional multivariable analysis further confirmed that adjuvant concurrent chemotherapy was independent prognostic factor for DFS, local recurrence-free survival and DMFS in cervical cancer patients with elevated SCC-Ag level.

In the present study, we found that preoperative SCC-Ag could act as a predictive marker for the use of adjuvant chemotherapy in cervical squamous cell carcinoma with intermediate-risk factors. Besides, increased pretreatment SCC-Ag levels was also a strong predictor of poor survival in cervical cancer patients and it has been widely used to predict the tumor recurrence after treatment [10, 16, 17]. In the study of Huang, et al., 188 patients with squamous cell carcinoma of the uterine cervix were retrospectively analyzed, with results showing that both SCC-Ag levels≥40 ng/mL (*p* < 0.001) and SCC-Ag levels of 10–40 ng/mL (*p* < 0.001) were significant factors for para-aortic lymph node recurrence. And the corresponding 5-year para-aortic lymph node recurrence rates were 84.8, and 27.5%, respectively, which just indicated that higher level of SCC-Ag caused higher rate of para-aortic lymph node recurrence [6]. In another study performed by Liu et al., one hundred ninety-seven cervical cancer patients who had received curative treatment with FIGO stage IB1 were included. Their data revealed that, among squamous cell carcinoma histology, patients with an Hb level less than 12 g/dl and a SCC-Ag level more than 3 ng/mL had worse oncologic outcomes [8]. Besides, some studies showed that elevated levels of SCC-Ag were significantly associated with lymph node metastasis, which was a major risk factor of impaired survival in cervical cancer patients [18]. But, the reported cut-off values of SCC-Ag level in predicting lymph node metastasis differed among the studies [19–21].

For patients with intermediate-risk factors who received adjuvant postoperative radiotherapy, the main treatment failure was distant metastasis [22]. This may be the possible reason of that adjuvant radiotherapy could only decrease local-regional recurrence, but failed to improve OS [23]. Adjuvant chemotherapy could decrease the rate of distant metastasis, thus the addition of chemotherapy to the treatment may be reasonable for cervical cancer patients after surgery. And it has been reported that adjuvant chemotherapy was effective in early stage cervical cancer with surgically confirmed intermediate risk factors [24]. However, few studies has directly compared the efficacy between adjuvant chemo-radiotherapy and adjuvant radiotherapy in cervical cancer with intermediate risk factors. We found that SCC-Ag can be used to guide the adjuvant concurrent chemotherapy. In details, for patients with high pre-treatment SCC-Ag level, adjuvant therapy should be administered to them due to the improvement in survival. While, in patients with low SCC-Ag level, adjuvant chemotherapy failed to improve the oncologic outcome. As we know, this new finding was the first to be reported and we suggested a novel clinical use of squamous cell carcinoma antigen. Besides, we also found that tumor size and deep stromal invasion were independent predictors of DFS and DMFS, which was in consistent with other study [25]. Our multivariate analysis showed that adjuvant chemotherapy was significantly associated with DMFS, indicating that patients who received adjuvant chemotherapy suffered less distant failure than those who did not. Based on the related discussion above, the possible explanation for our new finding were as follows: 1. high pre-treatment SCC-Ag level predicted high rates of recurrence and adjuvant chemotherapy was effective in cervical cancer patients with intermediate-risk factors; 2. Due to the poor survival in patients with high SCC-Ag level, adjuvant chemotherapy could significantly improve the oncologic outcome. However, in patients with low SCC-Ag level, the survival improvement brought by adjuvant chemotherapy may be little and not clinically significant because of the relatively favorable oncologic outcome in these patients who undergone adjuvant radiotherapy alone.

Some limitations were with our study. First, the selection bias could not be avoided because of the retrospective design of our study. But we found that most of the clinical variables were balanced between patients who did and did not have an elevated squamous cell carcinoma level. Secondly, the sample size in our work is relatively small. One of the reasons was that we only selected the patients with intermediate-risk factors, not including those with high-risk or no-risk factors. Besides, we chose the median pre-treatment level of SCC-Ag to divide all the patients into two group, which was based on the method adopted in other studies [26, 27].

## Conclusions

In conclusion, pre-treatment SCC-Ag can be a predictive marker for the use of adjuvant chemotherapy in cervical squamous cell carcinoma with intermediate-risk factors. Only those patients with high SCC-Ag can benefit from adjuvant chemotherapy. However, further larger-scale cohort studies are still warranted to prove this finding.

## Data Availability

The datasets used and/or analyzed during the current study are available from the corresponding author on reasonable request.

## Abbreviations

SCC-Ag: Squamous cell carcinoma antigen
DFS: Disease-free survival
OS: Overall survival
ELISA: Enzyme linked immunosorbent assay
CI: Confidence interval
HR: Hazard ratio
DMFS: Distant metastasis-free survival
LRFS: Local recurrence-free survival
CTV: Clinical target volume
